# Identification of conserved T cell epitopes and flanking amino acid mutants of endogenous retrovirus Gag antigen in nonobese diabetic mice

**DOI:** 10.1093/immhor/vlaf033

**Published:** 2025-08-25

**Authors:** Yang D Dai, Shuhui Li, Amanda Margosiak, Wen-Yuan Hu

**Affiliations:** HERV Laboratory, San Diego, CA, United States; Biomedical Research Institute of Southern California, Oceanside, CA, United States; Biosettia Inc., San Diego, CA, United States; Biomedical Research Institute of Southern California, Oceanside, CA, United States; Biosettia Inc., San Diego, CA, United States; Biosettia Inc., San Diego, CA, United States

**Keywords:** endogenous retrovirus, epitope, Gag, MHC, Qa-1

## Abstract

The interactions between endogenous retroviruses (ERVs) and major histocompatibility complex molecules may significantly influence autoimmune diseases due to their common roles in the evolution and development of the adaptive immune system. Notably, regions within the Gag antigens of a specific group of ERVs, similar to murine leukemia retroviruses, exhibit patterns of sequence conservation, variation, and mutation. One highly conserved peptide of Gag, p5-13 (VTTPLSLTL), binds with high affinity to a nonclassic major histocompatibility complex molecule, Qa-1, and is preferentially recognized by T cells enriched in the pancreas of nonobese diabetic (NOD) mice, which spontaneously develop autoimmune type 1 diabetes. Interestingly, deep sequencing analysis of the Gag genes expressed in NOD mice has revealed numerous mutations flanking the conserved Qa-1–binding sequences. This includes 1 epitope, p310-328, which contains both conserved and mutated residues that can elicit autoreactive T cells in NOD mice. A specific residue, D316, within this epitope accumulates multiple mutations as the disease progresses, leading to a reduction in the consensus score in sequence alignment at this position during the later stages of prediabetes. Consistently, the substitution of the D316 residue with a dominant mutant, G316, enhances the antigenicity of this epitope, stimulating autoreactive T cells in prediabetic NOD mice to release interferon-γ . Thus, sequence variants of ERV Gag antigens encode overlapping conserved and highly mutated epitopes that can be recognized by T cells and utilized for biomarker discovery.

## Introduction

Major histocompatibility complex (MHC) genes regulate resistance to infection by exogenous murine leukemia retroviruses (MuLVs)^1^ and susceptibility to multiple autoimmune diseases,^2^ indicating significant roles for specific antigens and their cognate T cells in the pathogenesis of both infectious and autoimmune responses. A common pathway between these responses involves endogenous retroviruses (ERVs). Numerous MuLV-like ERVs are present in the mouse genome.^3^ Research utilizing the Friend virus model has uncovered intrinsic interactions between exogenous and endogenous MuLV-like retroviruses,^4^ enhancing our understanding of complex antiviral innate and adaptive immunity.^5^ However, studies in autoimmune models present additional challenges, likely due to the absence of exogenous viruses analogous to human ERVs. Unfortunately, genetic linkage studies are typically indirect and often demonstrate weak associations,^6^ possibly influenced by epigenetic factors regulating ERVs. Nonetheless, foundational immunological studies have provided insights into the mechanisms connecting ERVs to autoimmunity, including the activation of innate or T-independent B cell responses by viral antigen products.^7^^,^^8^ Antibody responses to ERV antigens serve as valuable markers, although their role in autoimmune pathogenesis remains ambiguous.^9^ T cell responses to ERV antigens have been suggested based on a hypothetical “superantigen” activity, but there is no mechanistic evidence to support this.^10^ New evidence indicates alterations in the T cell repertoire responding directly to ERV antigens.^11^^,^^12^ However, the specific peptides responsible for these repertoire changes and the mechanisms by which they contribute to autoimmunity have not yet been thoroughly investigated.

Nonclassic MHC genes, also known as class Ib molecules, play a crucial role in immune regulation.^13^ These MHC class Ib molecules share structural similarities with conventional class Ia molecules; however, they exhibit significantly lower polymorphism, with few capable of encoding a functional MHC, as demonstrated by the Qa-1 molecule (H2-T23).^14^ Qa-1 has a stringent requirement for peptide binding, with 1 prominent Qa-1–binding peptide, the Qa-1 determinant modifier (Qdm) (AMAPRTLLL), derived from the conserved leader sequence of class Ia MHC genes.^15^ Recently, additional microbial and self-peptides have been identified that can stabilize Qa-1 molecules on the cell surface.^16^^,^^17^ Interestingly, despite its limited polymorphism, the Qa-1 locus has been mapped to encode an Rfv-2 gene that governs resistance to Friend virus–induced erythroleukemia,^18^ underscoring a vital role of Qa-1 in eliminating transformed cells. In the context of autoimmunity, Qa-1 is not typically considered a candidate gene for disease susceptibility; however, Qa-1–mediated antigen presentation is known to suppress autoimmune responses, potentially by promoting the development of CD8^+^ regulatory T cells.^19^^,^^20^ Notably, HLA-E, the human homolog of Qa-1, is upregulated in the islets of patients with type 1 diabetes (T1D),^21^^,^^22^ and its expression in pancreatic islets is likely influenced by local antiviral responses and interferon (IFN) signaling.

The NOD mouse is extensively used in autoimmunity research as a model that closely mimics human T1D. The islet-specific autoimmune response begins as early as 2 to 3 wk of age, coinciding with the early stages of islet development.^23^^,^^24^ This model is particularly well suited for studying the mechanisms by which ERVs trigger autoimmunity, considering the established role of MuLV-like ERVs in the islet autoimmune response.^25^ Similar to humans, there is a strong association with class II MHC genes in this model.^26^ T cell responses in the islets exhibit characteristics typical of an antigen-driven autoimmune response.^27–29^ Autoreactive T cells can be diabetogenic despite their relatively low affinity for common islet beta cell antigens.^30–32^ Additionally, the antiviral response, particularly involving type 1 IFNs and IFN-inducible genes, represents one of the earliest immune signatures observed during the islet inflammatory response in both mice and humans.^33^^,^^34^ ERV viral antigens likely play a critical role in triggering these antiviral responses and initiating subsequent autoimmunity.

In this study, we investigated the antigen Gag—the capsid protein—to elucidate the mechanisms modulating autoreactive T cells in NOD mice. Our previous research demonstrated that Gag serves as a significant T cell target, utilizing virus-like particles or exosomes as carriers for the Gag antigen.^35^^,^^36^ Notably, Gag-expressing exosomes are highly immunostimulatory, activating a substantial number of T cells to release IFN-γ following their injection into prediabetic NOD mice. Conversely, knocking down the Gag antigen using short hairpin RNA significantly decreases the level of induced IFN-γ.^37^ This suggests that Gag likely stimulates autoreactive effector T cells in vivo. Intriguingly, neither the Gag protein nor synthetic Gag peptides could replicate the exosomes' capacity to activate effector T cells, indicating the involvement of unique antigen processing and presentation mechanisms. Given that Gag is an ancient retroviral antigen that may have influenced the evolution of MHC genes, we explored the hypothesis that the nonclassical class Ib MHC molecule Qa-1 is involved in presenting the Gag antigen to elicit the T cell response. This study identified a Qa-1–restricted Gag peptide recognized by autoreactive T cells that expand in the pancreas of NOD mice during the early stages of prediabetes. Additionally, a second conserved peptide, which contains highly mutated residues in its flanking regions, can stimulate autoreactive T cells induced during the later stages. Ongoing efforts focus on revealing sequence variants at or near the conserved Qa-1–binding regions to uncover specific amino acid residues associated with autoimmune progression. Together, our findings imply a novel antigen-presenting pathway involving both nonclassic and classic MHC molecules, which may be crucial for breaking T cell tolerance to endogenous retroviral antigens such as Gag.

## Materials and methods

### Mice

NOD/ShiLtJ (NOD), C57BL/6 (B6), NOD.CB17-Prkdcscid/J (NOD.*scid*) mice, and the transgenic mouse NOD.BDC2.5, which expresses a diabetes-causing T cell receptor, were acquired from the Jackson Laboratory (Bar Harbor, ME). Experimental protocols utilizing these various mouse models were conducted with approval from the Institutional Animal Care and Use Committee of Biosettia Inc. and HERV Laboratory.

### Peptides

The peptides were synthesized by Biomatik, achieving over 90% purity and containing no amide groups. Peptides were dissolved in dimethyl sulfoxide at a concentration of 20 mg/mL for storage, and working stocks were prepared by dilution in water to 1 mg/mL.

### Islet and serum collection

Individual islets were hand-picked under a dissection microscope after digesting sliced pancreas with freshly prepared 0.46 mg/mL collagenase (Sigma-Aldrich) for 30 to 45 min at 37 °C until they were visibly softened and ready for disruption by manual shaking. For each mouse, 50 to 100 islets were collected. Retro-orbital eye bleeding was performed to collect blood samples, and 10-50 µL of serum was separated from each sample.

### Production of Gag-specific monoclonal antibodies and Sandwich enzyme-linked immunosorbent assay

Production of monoclonal antibodies (mAbs) specific for Gag was conducted by Biosettia. A recombinant Gag protein, islet.Gag150, derived from a Gag gene cloned from the islets of NOD mice, showed 99.88% identity to a reference ERV equence in chromosome 4 at positions 108152234 to 108153844 (reference mouse genome: GRCm38/GCA of C57BL/6J strain).^38^ This protein was utilized for immunization in conjunction with the Sigma Adjuvant System (MilliporeSigma). Spleens were used for fusions with PEG1500 (MilliporeSigma), and positive clones were screened for binding to 2 different Gag proteins: the one used for immunization, islet.Gag150, and the one cloned from Min6 insulinoma cells, Min6.Gag, which expresses a distant Gag gene with 84.84% homology to the reference but is identical to a published ERV sequence (GeneBank ID: DQ366147.1).^37^ The nucleotide sequences of the two Gag genes, islet.Gag150 and Min6.Gag, and the alignment of their protein sequences are shown in Fig. S1. Two rounds of subcloning were performed, and reactivity to the full Gag proteins and its subunits, including matrix and capsid proteins, was evaluated. One mAb, clone 9-27-45, which recognizes both full-length and capsid proteins of the two different Gag proteins (Fig. S2A), was selected as the Gag-capturing antibody in a Sandwich enzyme-linked immunosorbent assay (ELISA). A second cross-reactive mAb, clone 16-59-87, which recognizes only full Gag proteins but not matrix and capsid subunits (Fig. S2B), was selected as the detecting antibody. Avidin-HRP and substrate were used for colorimetric detection at a wavelength of 450 nm.

### Flow cytometry to detect Qa-1–binding T cells

IgFc-Qa-1 dimer and β2m recombinant proteins were produced by Biosettia. The Qa-1a gene (GenBank ID: L00606.1) was linked with the human IgG1 Fc region to form the Ig dimer. Protein expression was conducted in CHO cells using overexpression vectors, and purification was achieved using a Protein A column. To produce the recombinant β2m protein, the mouse β2m gene (GenBank ID: EDL28084.1) was tagged with an 8xHis sequence and inserted into expression vectors. The supernatant from transfected CHO cells was utilized for protein purification via a Ni-NTA system. For T cell staining, a mixture of IgFc-Qa-1, β2m, and peptide was incubated at room temperature for 2 h prior to use. The final concentrations of the mixture are 0.125 mg/mL for IgFc-Qa-1, 0.125 mg/mL for β2m, and 0.25 mg/mL for the peptide. Staining was performed using a 1:20 dilution of the mixture for 0.5 million cells at room temperature for 90 min. After washing, the cells were stained with fluorescent-labeled antibodies specific for hIgFc, B220, CD4, and CD8, and analyzed using a NovoCyte Advanteon flow cytometer.

### Cytokine analysis

Splenocytes from NOD mice (10^6^ cells/200 μL/well) were cultured with or without peptides for a duration of 48 h. The culture supernatants were subsequently harvested and stored at −20 °C until testing. IFN-γ-capturing beads were prepared by incubating 10^8^ Aldehyde/Sulfate Latex beads (4 µm) (Thermo Fisher Scientific; Cat# MT37338) with 100 µg/mL purified anti-IFN-γ capturing mAbs (Clone R4-6A2) (Invitrogen) in 500 µL of PBS for 2 h with rotation at room temperature, followed by blocking with 500 µL of 0.1 M glycine for 1 h. To detect IFN-γ, 100 µL of the supernatant was incubated with 10^6^ of the prepared capturing beads for 2 h, followed by detection using a 1:200 dilution of PE-labeled anti-IFN-γ (Clone XMG1.2) (Invitrogen). IFN-γ levels were quantified by measuring fluorescent intensity with a NovoCyte Advanteon flow cytometer.

### RNA purification, DNase treatment, and complementary DNA synthesis

For islets, total RNA was purified using an RNA extraction kit (Qiagen). Serum RNA was isolated using TRIzol reagent (Invitrogen) according to an industrial protocol, with the modification of adding glycogen. The purified RNA was treated with DNase I (New England Biolabs) at 37 °C for 30 min, followed by denaturation of the DNase I through the addition of 5 mM EDTA and incubation at 65 °C for 15 min. First-strand complementary DNA synthesis was conducted using SuperScript IV reverse transcriptase (Invitrogen) and a Gag-specific primer, 5′-TCTTCTAACCGCTCTAAC-3′, which targets a conserved C-terminal region of the ERV Gag gene.

### Amplicon deep sequencing analysis

Two sets of primers were used to produce amplicons of nt838-1197 (360 bp) and nt-95-376 (490 bp), which cover a conserved and variable region of the Gag gene, respectively. Primer sequences for nt838-1197 are 5′-GAGAAGCAGCGGGTGCTCC-3′ and 5′-GGACTGCCAGATGAATGACA-3′; primers for nt-95-376 are 5′-CGGTTTTTCGCCGAAACCG-3′ and 5′-AGAGCAGGGTAAAGGGCAGA-3′. Sequencing adaptors were added to these primers to label polymerase chain reaction amplicons: forward 5′-ACACTCTTTCCCTACACGACGCTCTTCCGATCT-3′ and reverse 5′-GACTGGAGTTCAGACGTGTGCTCTTCCGATCT-3′. Amplicons of the correct sizes were sliced from an agarose gel for purification using a DNA gel extraction kit (Qiagen). Amplicon sequencing and analysis were conducted as previously described by AZENTA, Inc.^39^ Matched sequences were aligned to identify different variants, open reading frames (ORFs), and mismatching nucleotides. ORF sequences were aligned using Jalview software 2.11.4.1 (The Barton Group, University of Dundee, UK) to calculate conservation and consensus for individual amino acid residues.

### Diabetes onset and statistical analysis

Blood glucose levels were measured twice weekly. Mice exhibiting 2 consecutive measurements exceeding 250 mg/dL within 1 wk were classified as diabetic. Descriptive statistics, including means and percentages, were employed to summarize the outcome measures. A *t* test was conducted to compare independent samples across different mouse strains and various age groups of NOD mice and *p*-values (*P*) were calculated.

## Results

### Expression of Qa-1 and Gag in islets of NOD mice

**Figure 1.**
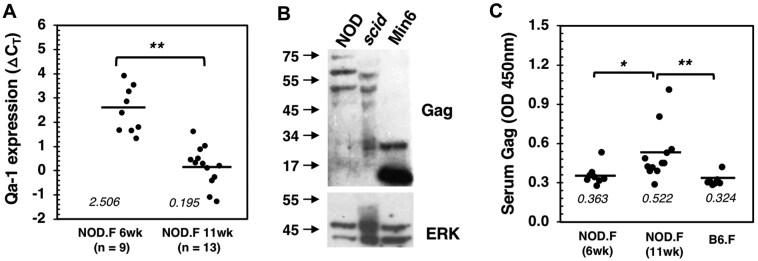
Expression of Qa-1 and Gag antigens in prediabetic NOD mice. (A) Qa-1 expression in the islets of 6-wk-old (n = 9) and 11-wk-old (n = 13) female NOD mice was quantified using real-time polymerase chain reaction and is presented as ΔCT (delta threshold cycles). Smaller ΔCT values indicate higher expression levels. ΔCT = CT.H2-T23(Qa-1) – CT.Rpl19(housekeeping). Each dot represents an individual mouse. Data were pooled from 2 independent experiments. (B) Approximately 300 islets collected from NOD or NOD.*scid* female mice (3–4 mice per group) were assessed for Gag protein expression through Western blot analysis utilizing a polyclonal anti-Gag goat serum for Gag detection. The insulinoma cell line Min6 was used as a positive control. (**C**) Detection of Gag antigen in serum samples. Serum from 6- or 11-wk-old female NOD mice or control B6 mice was diluted (1:10) in a sandwich ELISA using a pair of Gag-specific monoclonal antibodies, clones 9-27-45 and 16-59-87, for detection. Each dot represents an individual mouse. Similar results were obtained across three experiments. *P < 0.05; **P < 0.01. OD, optical density.

The expression of the Qa-1 gene in pancreatic islets was quantified using a commercial kit for real-time polymerase chain reaction analysis (Thermo Fisher Scientific; Cat# 4351372). Individual NOD female mice were analyzed, and age-differentiated groups, specifically 6- and 11-wk-old mice, were compared in order to assess Qa-1 expression in relation to autoimmune progression. A significant increase in Qa-1 expression was observed in the older age group, indicated by at least a 10-fold decrease in ΔCt values (Fig. 1A). Thus, Qa-1 is upregulated in the islets of prediabetic mice as the disease progresses.

The expression of Gag in pancreatic islets was detected using a Western blot assay, as previously described,^38^ using polyclonal goat serum specific for p15 of Gag (ATCC; Cat# CRL-1889). Multiple bands, including a full-length Gag protein (65 kDa) and degraded Gag fragments, were detected in islet samples from both NOD and NOD.*scid* mice (Fig. 1B). In contrast, the Gag fragments p30 and p15, products of digestion by retroviral protease, were highly expressed in Min6 cells, an insulinoma cell line known to express an ERV.^37^ This suggests a lack of the specific protease in NOD islets. This finding confirms the presence of Gag antigen in NOD islets, corroborating a previous observation made using a Gag-specific monoclonal antibody, R187,^40^ in immunohistochemistry staining.^38^

To detect Gag antigen in serum samples, we generated new mAbs specific for the Gag antigens expressed in the islets of NOD mice. One pair of mAbs was selected due to its high affinity for capsid protein and cross-reactivity with different Gag proteins. Serum samples were analyzed using this mAb pair in an ELISA to quantify Gag expression. A significant increase was observed in 11-wk-old NOD female mice compared with 6-wk-old NOD mice and T1D-resistant B6 mice (Fig. 1C), suggesting that the release of Gag antigen in serum may be associated with the progression of autoimmune responses in islets.

### Identification of Qa-1–restricted Gag peptides capable of modulating T cell responses

**Figure 2.**
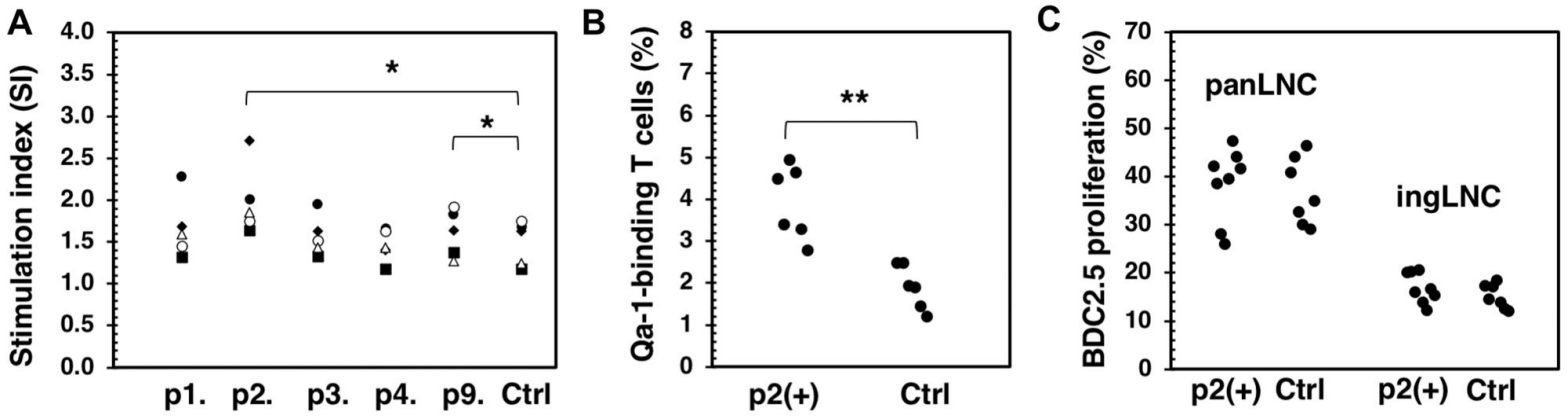
Regulating T cell responses by Qa-1–restricted Gag peptides. (A) Splenocytes were stimulated with anti-CD3/28 (1.0 µg/mL of each) in the presence or absence (control [Ctrl]) of Gag peptides shown (10 µg/mL) for 48 h. IFN-γ release in the supernatants was measured using a cytokine bead assay. Stimulation indexes were calculated as follows: IFN-γ in the presence of peptide/IFN-γ from anti-CD3/28 alone. (B) NOD female mice (6–8 wk old) were injected intraperitoneally with p2 peptides (10 µg/mouse) twice within 1 wk. Ctrl mice were age/sex matched, untreated mice. Qa-1–binding T cells in the spleens of the treated or Ctrl mice were subsequently measured. A recombinant IgFc-Qa-1 protein was used to stain Qa-1–binding T cells, with the percentage within the CD4^+^ population shown. Each dot represents an individual mouse. (**C**) CFSE-labeled BDC2.5 cells, obtained from transgenic mice, were transferred into the NOD mice treated as described in panel B. After 5 d, proliferating CFSE-low BDC2.5 T cells were quantified in the pancreatic or inguinal lymph nodes. Each dot represents 1 recipient mouse. *P < 0.05; **P < 0.01. ingLNC, inguinal lymph node cell; panLNC, pancreatic lymph node cell.

Using the IEDB epitope prediction tool (http://tools.iedb.org/main/), we identified several Gag peptides that bind to Qa-1. Table 1 lists these Gag peptides with high affinity for Qa-1. Among them, several peptides scored above 0.5, including the #p2 peptide (p5-13) VTTPLSLTL, which achieved the highest score of 0.701, compared with the control Qdm peptide (p1), AMAPRTLLL, which scored 0.795. To evaluate the potential influence of Qa-1–binding Gag peptides on T cell responses, we first performed in vitro T cell activation by stimulating splenic T cells with anti-CD3 and anti-CD28 in the presence of various Gag peptides. This in vitro polyclonal T cell response showed that the p2 peptide significantly increased IFN-γ release induced by anti-CD3 and CD28 (Fig. 2A). With the exception of p9, which promoted a modest increase in IFN-γ levels, the other Qa-1–binding peptides did not affect the T cell response to anti-CD3/28. Subsequently, we selected the p2 peptide for an in vivo assay. Notably, within 1 wk following the injection of the p2 peptide into NOD mice, the total number of Qa-1–recognizing T-helper cells increased by at least 2-fold (Fig. 2B), suggesting that the p2 Gag peptide indeed induces a T cell response in vivo. However, this increase in Qa-1–binding T cells induced by the p2 peptide had little to no impact on the pancreatic-specific proliferation of a diabetogenic T cell clone, BDC2.5 (Fig. 2C).^41^ The data indicate that Qa-1–binding T cells are unlikely suppressive for the islet-specific autoreactive T cells.

**Table 1.** Candidate Gag peptides binding to Qa-1.

Peptide ID	Name	Peptide (aa)	Length	Score	Rank
Qdm (Ctrl)	p1	AMAPRTLLL	9	0.795	0.01
Gag5–13	p2	VTTPLSLTL	9	0.701	0.04
Gag257–265		VLITHQPTW	9	0.678	0.06
Gag399–407		SAPDIGRKL	9	0.582	0.14
Gag100–108	p3	FVSPKLPPL	9	0.562	0.17
Gag120–129		AQPPSRSALY	10	0.523	0.23
Gag369–377		RLKEAYRRY	9	0.521	0.23
Gag137–146	p4	KSKPPKPQVL	10	0.504	0.27
Gag320–328	p9	TTTEGRNHL	9	0.383	0.6

aa, amino acid; Ctrl, control.

### Detection of Gag-specific T cells binding to Qa-1

**Figure 3.**
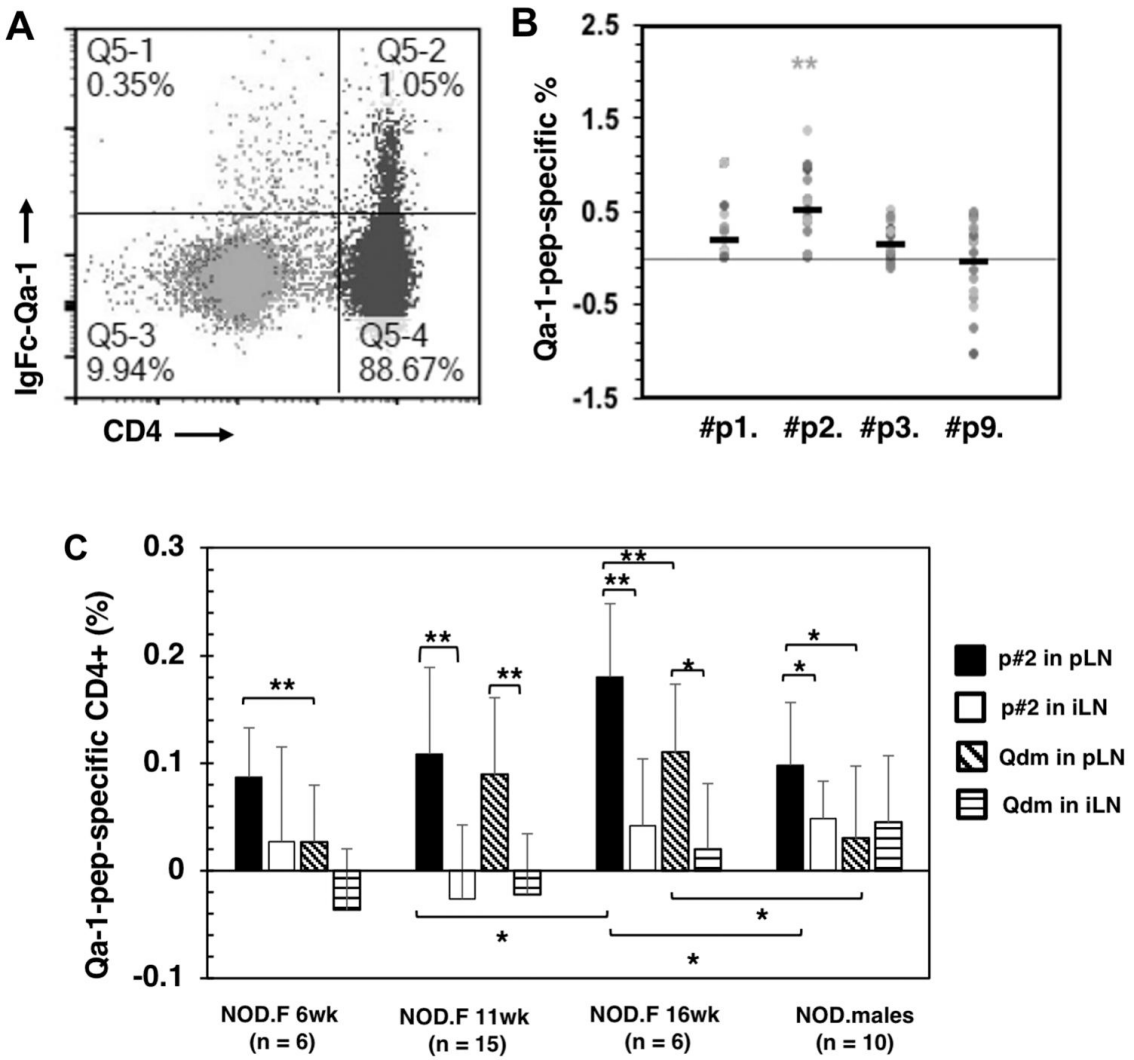
Detection of Qa-1–binding T cells recognizing Gag peptides. Gag-specific, Qa-1–binding T cells were detected using a mixture of recombinant IgFc-Qa-1 and β2m proteins (IgFc-Qa-1/β2m) in the presence or absence of Gag peptides. (A) Background staining of NOD pancreatic lymph node (pLN) cells with IgFc-Qa-1/β2m. Only B220-negative cells were analyzed. (B) Lymph node cells from NOD mice of different ages and breeding colonies were stained with IgFc-Qa-1/β2m along with various Gag peptides. Peptide-specific, Qa-1–binding CD4^+^ T cells were calculated as the percentage of IgFc-Qa-1/β2m/peptide–binding cells minus the percentage of the no-peptide control (IgFc-Qa-1 and β2m only). (C) Singel cells of pLNs or inguinal lymph nodes (iLNs) from NOD females (6, 11, and 16 wk old) and NOD male mice (8–10 wk old), purchased from the Jackson Laboratory, were stained with IgFc-Qa-1/β2m and Gag peptide p#2 (VTTPLSLTL) or the control Qdm peptide (AMAPRTLLL). A *t* test analysis was used to compare between groups as marked. *P < 0.05; **P < 0.01.

To assess the association between T cells recognizing different Gag peptides and the development of diabetes in NOD mice, we generated a Qa-1a IgFc-dimer protein, as NOD mice express the Qa-1a allotype. This allowed us to evaluate the frequency of T cells recognizing individual Gag peptides by staining T cells in the presence of β2m protein along with various Gag peptides. A relatively low background was achieved for the no-peptide controls by modifying the staining and gating methods (Fig. 3A). Although variation among different mice and breeding colonies is notable, ranging from 1% to 3% of total CD4^+^ T cells, the presence of Qa-1–binding peptides during the staining can result in an increased frequency of T cells that stain positive with the Qa-1a Fc-dimer. Notably, the p2 peptide emerged as the most effective at detecting Qa-1–binding T cells, despite exhibiting slightly lower affinity compared with the control Qdm peptide (Fig. 3B). Furthermore, a significant increase in Qa-1-p2–binding T cells was observed in the pancreatic lymph nodes of young NOD mice, while no increase was detected for Qa-1-Qdm–binding T cells (Fig. 3C). In contrast, p2-specific staining of T cells was not observed in the inguinal lymph nodes. These findings suggest that a Gag-specific response, particularly the p2-specific response, may contribute to the pancreas-specific expansion of Qa-1–binding T cells.

### Not all Qa-1–restricted Gag peptides are highly conserved

**Figure 4.**
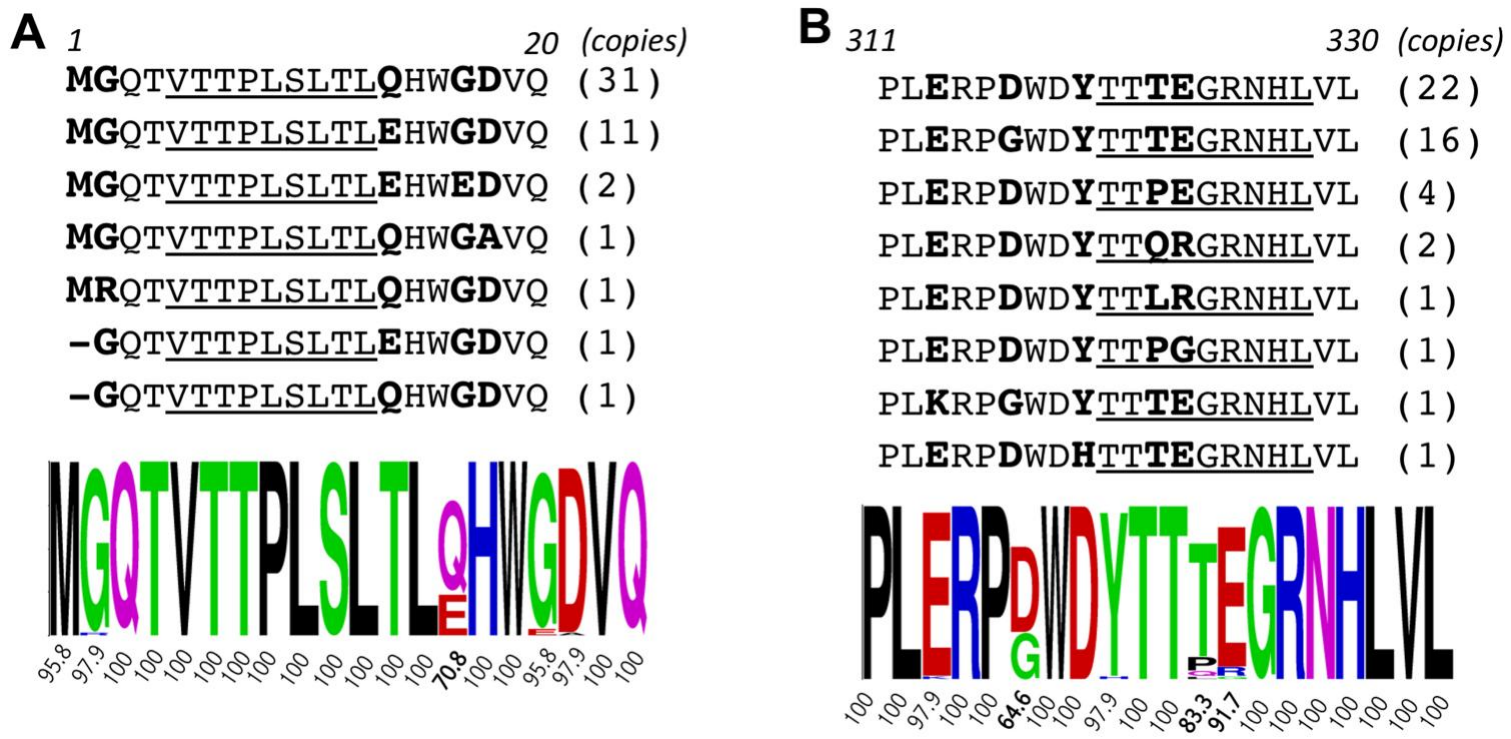
Identification of conserved and nonconserved Qa-1–Restricted gag peptides. An ERV Gag gene (GenBank ID: DQ366147) was utilized to search the mouse genome (GRCm38/mm10) for homologous sequences using the online BLAT genome search program (http://genome.ucsc.edu/cgi-bin/hgBlat). Only sequences encoding a complete ORF of Gag-like genes were selected. A total of 48 distinct ORFs were translated into amino acid sequences and used for alignment analysis. The p2 (A) and p9 (B) regions, extended at both the N- and C-termini, were examined to identify conserved and nonconserved amino acid residues among the 48 sequences. Predicted Qa-1–binding sequences are underlined, and nonconserved residues are in bold. An amino acid logo is plotted using the indicated consensus scores of the most abundant amino acid for each position.

To assess the conservation of Qa-1–restricted Gag peptides, we aligned Gag-like sequences identified in the mouse genome. A total of 48 full-length ORFs of Gag-like sequences homologous to MuLVs were identified. Many partial ORFs were also found in the genome but were excluded from this analysis due to the need for the capsid and the C-terminus of Gag in forming and releasing virus-like particles. By aligning the amino acid sequences of these 48 Gag proteins, we calculated consensus scores for each amino acid residue. Notably, the p2 sequence, p5-13: VTTPLSLTL, demonstrates remarkable conservation, with every residue aligning perfectly across all 48 Gag sequences (Fig. 4A). However, not all Qa-1–binding Gag peptides exhibit high levels of conservation. For instance, the p9 peptide, p320-328: TTTEGRNHL, shows significant variation; the two positions T322/E323 (TE) exhibit considerable differences, with 8 of 48 sequences containing mutant residues (Fig. 4B). This variation could affect binding to Qa-1 molecules. Interestingly, exogenous MuLV viruses, such as Friend and AKR strains, predominantly contain Q322/R323 (QR) instead of the TE. In fact, this region has been identified as a dominant factor influencing host susceptibility to the B- versus N-tropism of different MuLV viruses.^42^ However, the transition from TE to QR does not significantly alter the binding affinity to Qa-1 molecules. It is possible that other MHC molecules, but not Qa-1, could be the restriction factors for the viral tropism.

### NOD islets express distinct Gag sequence variants at the flanking residues of Qa-1–restricted peptides

**Figure 5.**
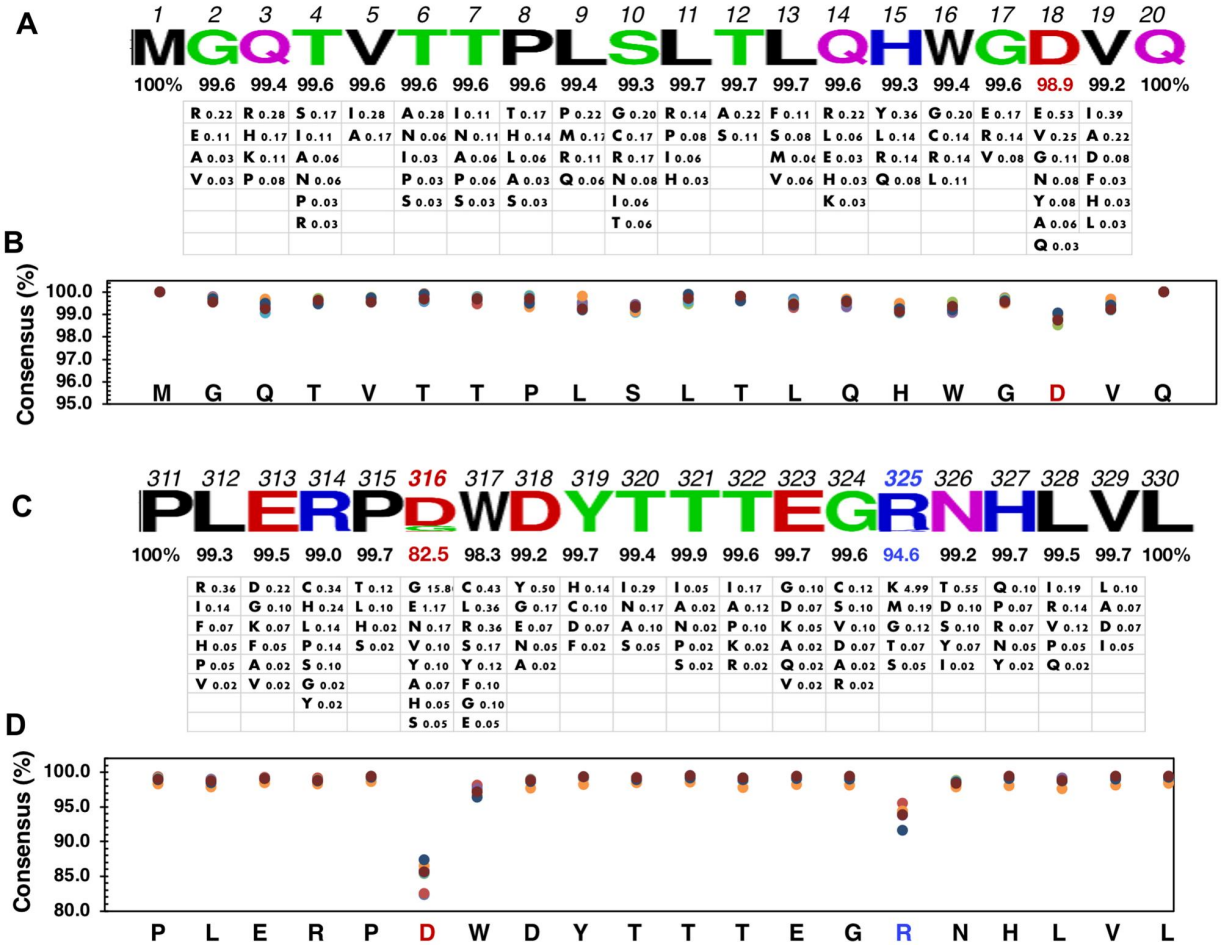
Deep sequencing analysis of Gag gene variants and ORFs in NOD Mice. Islet RNA samples were examined using targeted deep sequencing analysis to identify sequence variants and ORFs of Gag genes. (A) The p2 region is targeted using an amplicon (nt-95-376) for deep sequencing. ORFs were translated to protein sequences for alignment analysis using Jalview software to calculate consensus scores for individual amino acids at each position. Only positions 1 to 20 are shown, including the p2 sequence p5-13 (VTTPLSLTL). Scores for each consensus amino acid and their variants are listed under each position. (B) Eight NOD female mice at 6 wk of age were included to account for experimental variations. (C, D) The p9 region is targeted using an amplicon (nt838-1197) for deep sequencing and amino acid alignment analysis. Similarly, 8 islet samples from individual NOD females (6 wk old) were analyzed.

We previously observed that NOD islets express numerous Gag variants that are not fully homologous to any known genomic sequences. We interpreted this as evidence that post-transcriptional editing may occur in the Gag genes expressed in the islets during the autoimmune response.^39^ To determine whether mutational events in the islets affect the Qa-1 peptides, we conducted targeted deep sequencing analysis focusing on the p2 and p9 regions. For the p2 region (p5–13), we used an amplicon (nt-95-376, 490 bp) for deep sequencing analysis. Similar to the genomic sequence, the islet-expressing p2 sequences are strictly conserved (>99% consensus) (Fig. 5A), with a minor variable position at the D18 residue, which contains 1.1% variants differing from D18 (Fig. 5B). The p9 region (p320–328) is encompassed by a previously described deep sequencing amplicon (nt838-1197, 360 bp).^39^ Unlike the p2 region, the genomic p9 sequences (TTTEGERNHL) exhibit multiple variants at the TE positions, with consensus scores of 83.3% and 91.7%, respectively (Fig. 4B). However, this region becomes highly conserved for the Gag genes expressed in the pancreatic islets of NOD mice. Almost all islet-expressing Gag sequences contain the TE at the 322/323 positions, with consensus scores of 99.6% and 99.7%, respectively (Fig. 5C). Therefore, the p9 peptide is also highly conserved for islet-expressing Gag, with the exception of one residue, R325, which shows a slightly lower consensus score of 94.6%. Surprisingly, an adjacent residue, D316, flanking the N-terminus of p9, has a much lower consensus score of 82.5% (Fig. 5C). This highly variable residue, D316, was confirmed by analyzing eight individual NOD mice (Fig. 5D). Two major amino acid substitutions—G316 and E316—were identified at frequencies of 15.8% and 1.17%, respectively, along with several other substitutions at frequencies lower than 1%, including N, V, Y, A, H, and S residues. The data suggest that the flanking residues of Qa-1 peptides, particularly the N-terminal flanking residue D316 of the p9 peptide, may contain mutated residues.

### Sequence variants of the flanking residues are associated with autoimmune responses

**Figure 6.**
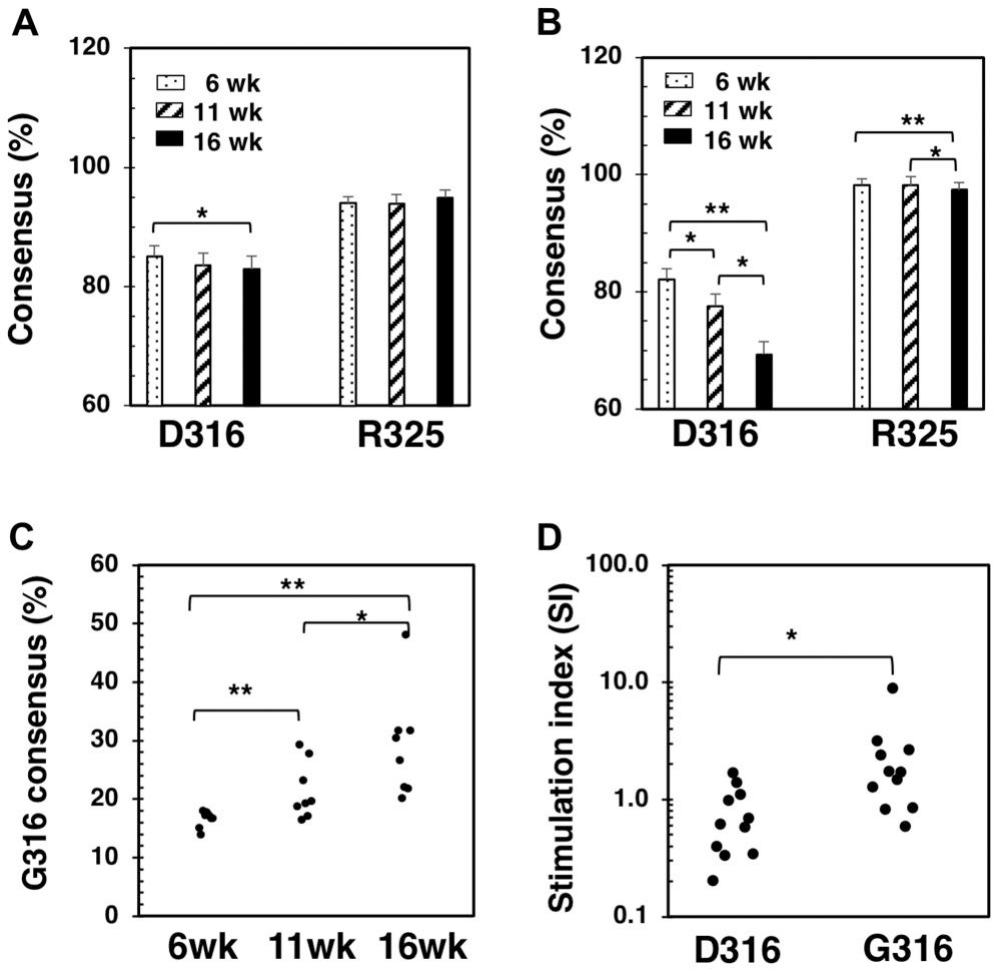
Evaluating Gag variants in autoimmune responses and T1D development. (A, B) Consensus levels of specific amino acid residues can serve as disease biomarkers for monitoring autoimmune progression. Pancreatic islets (A) and sera (B, C) from NOD female mice of different ages (n = 8 per age group) were analyzed using the amplicon nt838-1197. (B) Average consensus scores at positions D316 and R325 of the p9 region are shown. (C) Consensus scores for G316 of individual mice at different age groups (n = 8 per group) are shown. A *t* test was performed to compare different age groups. (D) NOD female splenocytes (8–10 wk old) were stimulated in vitro using synthetic peptides (10 µg/mL) p310 to p328, which either contain D316 or G316. IFN-γ release in the supernatants was measured in a cytokine bead assay, and stimulation indexes were calculated as follows: IFN-γ with peptide/IFN-γ of medium control. Each dot represents a single mouse. *P < 0.05; **P < 0.01.

To investigate whether the highly variable residues contribute to autoimmune responses, we examined the patterns of mutant generation at different stages of diabetes in NOD mice. We analyzed the D316 and R325 positions for the sequence variants expressed in the islets of female NOD mice at 3 ages: 6, 11, and 16 wk (8 mice per age group). Notably, the consensus levels for the D316 position decreased significantly as the disease progressed, from 85.12% at 6 wk to 82.86% at 16 wk (Fig. 6A). This decrease was even more pronounced when serum samples from the same mice were analyzed, with consensus levels starting at 82.15% for the 6-wk-old mice, followed by 78.05% for the 11-wk-old mice, and dropping further to 71.19% in the sera of 16-wk-old mice (Fig. 6B). A minor decrease in the consensus score for the R325 residue was also observed. A dominant mutant at the D316 is G316, whose consensus scores significantly increased as the mice aged (Fig. 6C). Thus, the p9 region appears to be targeted for an increased number of mutants at the flanking residues, particularly the D316, that correlate with disease progression. To determine whether substitutions of the flanking residues could alter T cell responses, we tested synthetic peptides representing specific mutations of the p9 region to stimulate T cells in NOD mice. We observed a significant increase in IFN-γ release by NOD splenocytes when substituting a D316 peptide (p310–328/*D316*: FPLERP*D*WDYTTTEGRNHL) with a G316 peptide (p310–328/*G316*: FPLERP*G*WDYTTTEGRNHL) for stimulation (Fig. 6D). This suggests that the G316 peptide is more antigenic than the D316 peptide in activating effector T cells in NOD mice.

## Discussion

T cells that recognize the same antigen or even the same epitope may function differently in terms of immune regulation or pathogenesis.^43^ This is exemplified by T1D-associated beta cell antigens such as GAD65^44^^,^^45^ and insulin.^46^ Inducing islet-specific regulatory cells may suppress autoimmune responses; however, the epitopes and T cell repertoires for each of the common islet antigens appear insufficient to alter the course of T1D. In contrast, ERV antigens differ from islet antigens in terms of antigen abundance and their corresponding repertoires. Many ERVs contain partial or complete ORFs that code for retroviral antigens, and some may produce virus-like particles, particularly newly incorporated ERV members, such as the muLV-like ERVs expressed in the islets of NOD mice^25^ and virus-like particles produced by the insulinoma cell line Min6.^37^ Notably, ERV antigens have been suggested to function as superantigens, stimulating a large number of different T cells.^11^^,^^12^ The potential superantigenic activity of ERV antigens could overcome the limitations posed by islet-specific antigens and their insufficient repertoires. Further studies are needed to identify and characterize ERV epitopes and the responding T cells to determine whether and how ERV antigens modulate autoimmune responses.

To address the complexity of ERV sequences, we employed deep sequencing to analyze numerous ERV sequence variants, with the goal of identifying both highly conserved epitopes and variable amino acid residues. We chose the Gag antigen due to its greater conservation compared with other retroviral antigens and the supporting evidence from our data regarding its critical role in inducing autoreactive T cells.^37^^,^^38^ Our hypothesis proposed that highly conserved peptides would preferentially induce regulatory responses, while variable amino acid residues could generate agonistic high-affinity peptides to enhance effector T cell function. We focused on peptides restricted by a nonclassic MHC, Qa-1, using an MHC dimer method. This approach is based on the idea that antigen presentation by the Qa-1 molecule may have been evolutionarily significant in combating infections from ancient retroviruses millions of years ago. We successfully identified a dominant Qa-1–binding peptide, p2, which exhibits a high affinity comparable to the known Qdm peptide binding to the Qa-1 molecule. Notably, T cells recognizing this highly conserved Gag peptide are enriched in the pancreases of NOD mice, more so than those recognizing the Qdm peptide. The identification of the p2 epitope suggests that this region is a target for T cells.

The discovery that specific flanking residues, such as D316, contain mutations associated with disease progression presents an opportunity to identify autoreactive T cells that may be converted into effectors by interacting with the mutants. In fact, we and others have previously shown that flanking residues not involved in MHC binding can enhance or inhibit T cell activation depending on specific alterations and T cell clones.^44^^,^^47^^,^^48^ Whether T cells that interact only with conserved residues of Gag play a regulatory role or act as polyspecific autoreactive T cells^49^ requires further study. Intriguingly, the p2 epitope is predicted to have a stronger affinity for Qa-1 than the p9 peptide; however, it does not contain highly variable flanking residues, and we could not identify an altered synthetic peptide of the p2 region that can stimulate NOD splenocytes to release IFN-γ, as achieved by the p9 region peptide G316 (p310–328). Nevertheless, p2 appears to be the best peptide for detecting Qa-1–binding T cells in the pancreas. One possible mechanism is intramolecular epitope spreading,^50^ in which the dominant peptide p2 is recognized early, while the weaker Qa-1–binding peptide p9 may function as a cryptic peptide with potential for higher pathogenicity. However, because Qa-1 is much more stringent in peptide binding than class II MHC molecules, our approach, beginning with Qa-1, would facilitate the mapping of dominant epitopes and future investigations into how ERV antigens and epitopes participate in T cell–mediated autoimmune responses. The variable residues are likely to be attractive T cell targets, potentially by binding to flexible MHC molecules like I-A^g7^,^51^ while the conserved ERV epitopes may be preserved for Qa-1 binding, which may be favored for inducing regulatory cells. The choices and/or interactions in presenting the various p2 and p9 peptides by Qa-1 and I-A^g7^ molecules might be key in determining the finely tuned balance between antigen-specific regulatory and pathogenic T cells.

Finally, the targeted amplicon deep sequencing technique offers a novel solution for addressing complex ERV sequences, enabling the identification of conserved versus variable regions that could guide epitope discovery. It is important to note that the ORFs used in this technique are not “real” ORFs, as we only targeted a partial region of Gag; other regions may contain stop codons that prevent Gag protein translation. Longer reads would be beneficial for identifying true ORFs that encode full-length proteins, likely key antigens responsible for the T cell response. Additionally, by focusing on these “real” ORFs, we could significantly enhance sensitivity regarding disease biomarkers and the identification of key residues that contribute to T cell activation, along with any potential clinical relevance.

## Supplementary Material

vlaf033_Supplementary_Data

## Data Availability

The deep sequencing data will be available through specific request by sending an email to the Principal Investigator, Y.D.D. They will be available for request at least 5 years after the publication of the study.
