# Supplementary material for: Identification of conserved T cell epitopes and flanking amino acid mutants of endogenous retrovirus Gag antigen in nonobese diabetic mice
Source: Immunohorizons. 2025 Aug 25;9(9):vlaf033. doi: 10.1093/immhor/vlaf033 (PMC12377906; doi:10.1093/immhor/vlaf033)
Supplement: vlaf033_Supplementary_Data [file vlaf033_supplementary_data.pdf]

A

```
atgggacagaccgtaactacccctctgagtttaaccttgcagcactgg
ggagatgtccagcgcattgcatccaaccagtctgtggatgtcaggaag
aggcgctggattaccttctgttccgctgaatggccaactttcaatgtg
ggatggcctcaggatggtactttcaatttaagtattatctctcaggtt
aagtctagagtgttttgtcctggtccccacggacacccagatcaggtc
ccatatatcgtcacctgggaggcacttgcctatgacccccctccgtgg
gtcaaaccgtttgtgtctcctaaacttcctcccttgccgacagctccc
gtcctcccgcccggtccttctgcgcaacctccgtcccgatctgccctt
taccctgcccttacccccctctataaagtccaaacctcctaagccccag
gttctccctgatagcggcggacccctcattgaccttctcacagaggac
cccccgccgtacagagcacaaccctcctcctctgccagggagaacgac
gaagaagaggcggccaccacctccgaggtttcccccccttctcccatg
gtgtctcgactgcggggaaggagagaccctcccgcagtgggctccacc
acctcccaggcatttccactccgcatgggggggagatggccagcttcag
tattggccgttttcctcttcggatttatacaattggaaaaataataac
ccttccttttctgaagatccaggtaaattgacggccttgattgagtcc
gtcctcatcacccaccagcccacctgggacgactgtcagcagttgttg
gggaccctgctgaccggagaagaaaagcagcgggtgctcctagaggct
agaaaggcagtccggggcaatgatggacgccccactcagttgcctaat
gaagtcaatgctgcttttccccttgaacgccccggttgggattacacc
actacagaaggtaggaaccacctagtcctctatcgccagttgctctta
gcgggtctccagaacgcgggcagaagtcccaccaatttggccaaggta
aaagggataacccagggacctaatgagtctccctcagccttttttagag
agactcaaagaggcctatcgcaggtacactccttatgaccctgaggac
ccagggcaagaaaccaatgtgtctatgtcattcatctggcagtctgcc
ccggatatcgggcgaaagttagagcggttagaagatttaaagagcaag
accttaggagacttagtgagggaagctgaaaagatctttaataagcga
gaaaccccggaagaaagagaggaacgtatcaagagagaaacagaggaa
aaagaagaacgccgtagggcagaggatgagcagagagagaaagaaagg
gaccgcagaagacatagagagatgagcaagctcttggccactgtagtt
attggtcagagacaggatagacagggggggagagcggaggaggccccaa
cttgataaggaccaatgcgcctactgcaaagaaaagggacactgggct
aaggactgcccaaagaagccacgagggccccgaggactgaggccccag
acctccctcctaaccttaggtgac
```

B

```
atgggacaaaccgtaaccacccctctgagtctgaccctagaacactgg
gaagatgtccagcgcatcgcgtccaatcagtccgtagatgtcaagaag
agacgctgggtcaccttctgctctgccgagtggccaactttcggtgta
gggtggccacaagatggtacttttaacttggacattattctacaggtt
aaatctaaggtgttctctcctggtccccacggacacccggatcaggtc
ccatatattgtcacctgggaggctattgcctatgaacccccctccgtgg
gtaaaacctttgtctctcccaaactctcccccctctccaaccgctccc
atcctcccatccggtccttcgacccaacctccgccccgatctgccctt
taccctgctcttacccccctctataaaacccagaccttctaaacctcag
gttctctccgataatggcggacctctcattgaccttctctcagaagac
cctccgccgtacggaggacagggaccgtcctcctctgacggagatggc
gacagagaagaggccacctccacttctgagattcctgccccctctccc
atagtgtctcgcctgcggggcaaaagagaccccccgcggcagattcc
accacctctcgggctttcccactccgtttgggggtaatggtcagttg
cagtactggccgttttcctcctctgatctatataactggaaaaataat
aatccttccttctctgaggatccaggtaaactgactgcattgattgaa
tctgtcctcaccacccaccagcccacctgggatgattgccagcaatta
ttagggactctgcttaccggggaggagaagcagcgggtgctcctggaa
gcccgaaaagctgtccggggcaacgatgggcgccccacccaactgccc
aacgaggttgacgctgcttttccccttgaacgtcccgattgggattac
accacccaaagaggtaggaaccacctagttctctatcgccagttgctt
ttagcgggtctccaaaacgcgggccgaagccccaccaatttggccaag
gtaaaaggaataacccagggacctgatgagtccccctcagccttccta
gagagactcaaggaagcctatcgcagatacactccttatgatcctgag
gaccctgggcaagaaacgaatgtatctatgtcattcatctggcagtcc
gctccagacattggtcgaaagttagagcggttagaagacttaaaaagt
aaaactttaggagatttagtgagagaagccgaaaggatctttaataaa
agagaaactccagaagaaagagaagagcgcgttaggagggagacagag
gaaaaggaagagcggcgtagggcagaagaggagcagaaagagaaagag
agggaccgtaggagacatagagaaatgagcaaactttggccaccgta
gttagtggacagagacaggatagacagggggggagagcgaaggaggccc
caacttgacaaggatcaatgtgcctactgtaaagaaaaaggacactgg
gctaaagactgcccaaagaagccacggggtccccgaggaccgcgaccc
cagacctccctcctgactttagacgac
```

C

```
1     MGQTVTTPLSLTLQHWGDVQRIASNQSVDVRKRRWITFCSAEWPTFNVGWPQDGTFNLSIISQVKSRVFCPGPHGHPDQV   80
1     MGQTVTTPLSLTLEHWEDVQRIASNQSVDVKKRRWVTFCSAEWPTFGVGWPQDGTFNLDIILQVKSKVFSPGPHGHPDQV   80

81    PYIVTWEALAYDPPPWVKPFVSPKLPPLPTAPVLPPGPSAQPPSRSALYPALTPSIKSKPPKPQVLPDSGGPLIDLLTED  160
81    PYIVTWEAIAYEPPPWVKPFVSPKLSPSPTAPILPSGPSTQPPPRSALYPALTPSIKPRPSKPQVLSDNGGPLIDLLSED  160

161   PPPYRAQ-PSSSARENDEEEAATTSEVSPPSPMVSRLRGRRDPPAVGSTTSQAFPLRMGGDGQLQYWPFSSSDLYNWKNN  239
161   PPPYGGQGPSSSDGDGDREEATSTSEIPAPSPIVSRLRGKRDPPAADSTTSRAFPLRLGGNGQLQYWPFSSSDLYNWKNN  240

240   NPSFSEDPGKLTALIESVLITHQPTWDDCQQLLGTLLTGEEKQRVLLEARKAVRGNDGRPTQLPNEVNAAFPLERPGWDY  319
241   NPSFSEDPGKLTALIESVLTTHQPTWDDCQQLLGTLLTGEEKQRVLLEARKAVRGNDGRPTQLPNEVDAAFPLERPDWDY  320

320   TTTEGRNHLVLYRQLLLAGLQNAGRSPTNLAKVKGITQGPNESPSAFLERLKEAYRRYTPYDPEDPGQETNVSMSFIWQS  399
321   TTQRGRNHLVLYRQLLLAGLQNAGRSPTNLAKVKGITQGPDESPSAFLERLKEAYRRYTPYDPEDPGQETNVSMSFIWQS  400

400   APDIGRKLERLEDLKSKTLGDLVREAEKIFNKRETPEEREERIKRETEEKEERRRAEDEQREKERDRRRHREMSKLLATV  479
401   APDIGRKLERLEDLKSKTLGDLVREAERIFNKRETPEEREERVRRETEEKEERRRAEEEQKEKERDRRRHREMSKLLATV  480

480   VIGQRQDRQGGERRRPQLDKDQCAYCKEKGHWAKDCPKKPRGPRGLRPQTSLLTLGD   536
481   VSGQRQDRQGGERRRPQLDKDQCAYCKEKGHWAKDCPKKPRGPRGPRPQTSLLTLDD   537
```

Figure S1. *Reference sequences for ERV Gag genes.* (**A**) The islet.Gag150 gene was cloned from the islets of NOD mice. (**B**) The Min6.Gag gene was cloned from the Min6 insulinoma cells. (**C**) Alignment of amino acid sequences of the islet.Gag150 (top) and Min6.Gag (bottom) proteins.

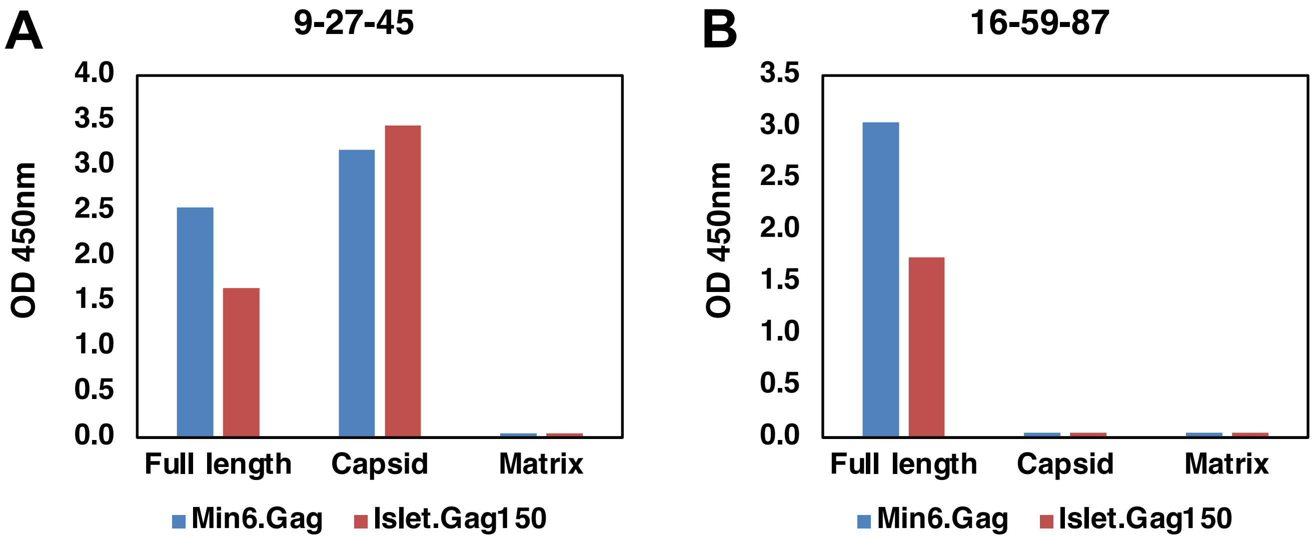

Figure S2. *Binding to Gag-specific mAbs to recombinant Gag proteins and subunits in ELISA assays.* Wells were coated with 4 µg/ml of Neutravidin, followed by incubation with 1 µg/ml of biotin-labelled recombinant Gag proteins (full-length) or subunit Capsid and Matrix. The binding of mAbs was performed at 1 µg/ml and detected with donkey anti-mouse IgG-HRP and substrate. The gene sequences used for protein expression (lentiviral expression in 293T cells) are derived from the islet.Gag150 or Min6.Gag genes.
